# Time trends in incidence of pilonidal sinus disease from 1996 to 2021: A Danish population‐based cohort study

**DOI:** 10.1111/codi.17227

**Published:** 2024-11-03

**Authors:** Ida Kaad Faurschou, Rune Erichsen, Dietrich Doll, Susanne Haas

**Affiliations:** ^1^ Department of Clinical Epidemiology Aarhus University Hospital and Aarhus University Aarhus Denmark; ^2^ Department of Surgery, Pilonidal Disease Center Randers Regional Hospital Randers Denmark; ^3^ Department of Clinical Medicine Aarhus University Hospital and Aarhus University Aarhus Denmark; ^4^ Department of Procto‐Surgery and Pilonidal Sinus, St. Marienhospital Vechta Academic Teaching Hospital of the MHH Hannover Vechta Germany

**Keywords:** age distribution, epidemiology, gender distribution, incidence, national health care data registries, pilonidal disease, pilonidal sinus, pilonidal sinus disease, population‐based, sex distribution

## Abstract

**Aim:**

Pilonidal sinus disease (PSD) is a common condition, but no data on disease occurrence exist outside highly selected settings. The aim of this study was to assess time trends in the incidence of PSD in a nationwide setting.

**Method:**

Using data from nationwide Danish registries, we identified 48 247 patients recorded with diagnostic or surgical procedure codes representing PSD between 1996 and 2021. We stratified by sex and computed the age‐adjusted and age‐specific incidence rate, comparing 5‐year intervals with the incidence rate ratio (IRR).

**Results:**

The overall incidence of PSD increased from 26.1 to 39.6/100 000 person‐years (PY) from the period 1996–2000 to the period 2016–2021 (IRR 1.52, 95% CI 0.78–2.94). The incidence increased from 35.8 to 56.9/100 000 PY (IRR 1.59, 95% CI 0.52–4.89) in male patients and from 16.4 to 22.5/100 000 PY (IRR 1.37, 95% CI 0.68–2.76) in female patients. The peak of age‐specific incidence was 215.7/100 000 PY (95% CI 206.1–245.4) among 20‐year‐old men and 107.9/100.000 PY (95% CI 100.0–114.0) among 18‐year‐old women. Over the study period, the median age at first hospital contact decreased from 27 years [interquartile range (IQR) 22–34 years) to 25 years (IQR 20–34 years) in men but remained stable around 23 years (IQR 18–32 years) in women. However, for both sexes, the highest increase in incidence was seen in early adolescence.

**Conclusion:**

The incidence of PSD has increased significantly over the last decades. The increase is driven primarily by men and boys, with the highest increase in incidence seen in early adolescence. The increased burden of disease is not reflected in the literature, and more studies are warranted to understand the drivers of this development.

**What does this paper add to the literature?**

This study is the first to examine pilonidal sinus disease in a nationwide population‐based setting. The incidence is increasing in both sexes while the age of debut is decreasing. The development is primarily driven by young men, even though female patients dominate young adolescents.

## INTRODUCTION

Pilonidal sinus disease (PSD) is primarily located in the natal cleft and is characterized by the entrapment of loose hair creating and/or entering the pathognomonic midline pits. This process triggers an inflammatory response, which may result in the development of cysts, sinuses or abscesses [1, 2]. PSD causes pain, discharge and smell, and often requires several surgeries. It usually affects teenagers and young adults and may impact the quality of life significantly [3, 4]. Patients with PSD are commonly seen in the clinic but are often underprioritized both in practice and in research, which is evident from the missing consensus due to limited scientific strength behind existing recommendations [5, 6].

The incidence of PSD has been insufficiently studied and originates from specific cohorts, while population‐based data are lacking. However, studies from Germany have suggested an increase in PSD incidence based on data from military registries and inpatient settings [7, 8]. Using data from the German military registries, a study showed that the incidence rate of PSD increased from 0.3 per 1000 persons in 1985 to 2.4 per 1000 persons in 2007 [7]. Based on German data on inpatient admissions for surgical treatment of PSD, the incidence in the German population is estimated to have increased from 30/100 000 inhabitants in 2000 to 48/100 000 inhabitants in 2012, though only presented as sole numbers in a German guideline [9]. Another German study confirmed this increase in PSD incidence based on inpatient data from 2005 to 2017 [8]. However, since PSD patients often undergo multiple admissions, the number of hospital admissions is not directly equal to the number of patients with PSD and may falsely increase the incidence. Furthermore, the increase may also be due to changes in treatment practices. Apart from incidence, the study also examined the sex proportion and showed a higher rate of inpatient admissions for PSD among male patients compared with female patients [8]. This finding aligns with a meta‐analysis reporting that women represent approximately 21% of PSD cases, with significant regional variations ranging from 7% to 39% [10]. However, the sex and age distribution of PSD is notably lacking in the literature, as the disease is often studied in military cohorts representing a specific demographic group mainly composed of young men or in selected settings, for example only elective PSD.

Using longitudinal data from the population‐based Danish health registries, we aimed to estimate time trends in the incidence of PSD from 1996 to 2021 through a comprehensive population‐based study. Additionally, we aimed to determine the age‐ and sex‐specific distribution of PSD within the population.

## METHOD

### Design and setting

We conducted a population‐based study using nationwide Danish registry data. Denmark has a tax‐funded healthcare system ensuring free healthcare for all residents [11]. The vast majority of treatments are delivered in the public system, and Danish citizens are primarily referred to public hospitals and only rarely directly to private clinics. Private hospitals and outpatient clinics are limited in number, contributing only 2.2% to overall hospital activity in 2010. They only handle selected procedure types and often only deal with simple illnesses. Additionally, since 2003, private hospitals and outpatient clinics have been required to adhere to the same reporting rules as public hospitals [12].

Data sources for this study included the Danish Civil Registration System [13] and the Danish National Patient Registry (DNPR) [12]. The Danish Civil Registration System contains data about the vital status and migration of all residents in Denmark. All residents are assigned a unique 10‐digit personal identification number at birth or immigration, which permits accurate linkage of individual‐level data across various health records [13, 14]. The DNPR covers all Danish hospitals and includes the personal identification number, dates of admission and discharge, procedure codes and up to 20 discharge diagnostic codes given by the discharging physician. The DNPR was established in 1977 with complete coverage since 1978 and contains records of all inpatient hospital contacts. From 1995 onwards, the DNPR has also recorded all outpatient and emergency room contacts [12].

The DNPR contains diagnostic codes recorded using the World Health Organization's International Classification of Diseases 8th revision (ICD8) from 1971 to 1993 (used to include a wash‐out period) and the 10th revision (ICD10) from 1994 onwards. Surgical procedure codes were Danish surgical codes from 1971 to 1995 and the Danish version of the NOMESCO (The Nordic Medico‐Statistical Committee) Classification of Surgical Procedures from 1996 onwards. Diagnostic codes consisted of an overall, an abscess‐forming and a nonabscess‐forming code. The procedure coding from 1996 to 2010 included a code for incision and excision procedures, but it was updated in 2010 to differentiate between specific excision procedures, for example pit‐pick and cleft (Table S1).

In 2019, substantial structural changes were made to data reporting and organization in the DNPR, improving the registration of patient flow.

### Study population

The study population comprised all citizens with a diagnostic or procedure code specific to PSD recorded in the DNPR between 1 January 1996 and 31 December 2021 (see Table S1 for specific codes). Using the unique 10‐digit personal identification number, we linked data from all patients to the Danish Civil Registration System. The first contact with a PSD diagnosis or procedure code was defined as the index contact. Patients were categorized by sex, age group at index contact (0–12, 13–15, 16–20, 21–25, 26–30, 31–35, 36–40, 40+ years) and disease type (abscess or nonabscess‐forming PSD). The study period was grouped into 5‐year intervals. To ensure that only index diagnoses were included, we implemented a wash‐out period from 1978 to 1995, excluding all patients with an index contact prior to 1996. As the DNPR encompasses all hospitals in Denmark, its data represent the entire population. Consequently, there is no selection bias related to specific patient groups and minimal loss to follow‐up, as only patients relocating outside Denmark are lost. Age and sex were extracted from the unique 10‐digit personal identification number, preventing missing data on these variables.

### Statistical analysis

#### Age‐standardized incidence rate

We computed the annual incidence rate as the number of newly diagnosed PSD cases divided by the population size in each of the years 1996–2021. Data were stratified by sex and standardized to the age distribution of the 2018 population. Furthermore, we calculated the incidence rate in 5‐year intervals and age‐standardized to the age distribution in the period 2016–2021. To evaluate the time trend in incidence rates, we computed the incidence rate ratio (IRR) and incidence rate difference (IRD) for each 5‐year period with the first period as reference.

#### Age‐specific crude incidence

To evaluate the change in age‐specific incidence, we calculated the crude age‐specific incidence rate by dividing the annual incidence rates of newly diagnosed PSD cases for each age group by the corresponding population size within the same age group for 1996–2021. To assess changes in age‐specific incidence, we stratified by sex and age groups and computed the IRR and IRD as defined above.

#### Validation of diagnostic coding

We randomly selected 20–40 patients with a discharge diagnostic code specific for PSD (50% nonabscess and 50% abscess code) in each of four Danish hospitals (Randers, Viborg, Gødstrup and Horsens), summing up to a total of 110 patients. For these patients, medical records were retrieved and reviewed by a physician (I. K. Faurschou, S. Haas, R. Paramasivam and P. Dalsgaard) (see Acknowledgements). The validation was approved by the hospital management in each of the four hospitals. In total, 108 out of 110 patients with a discharge diagnosis of PSD had PSD confirmed by the medical record review corresponding to a positive predictive value (PPV) of 98.2 (95% CI 92.9–99.7).

Analyses and illustrations were performed using R Software, version 4.3.2 [15]. In accordance with Danish legislation, registry‐based studies do not necessitate informed consent or approval from an ethics committee. The study was reported to Aarhus University and received the registration number 2624. The study adhered to the STROBE reporting guideline [16] of the EQUATOR network.

## RESULTS

### Descriptive data

**FIGURE 1:**
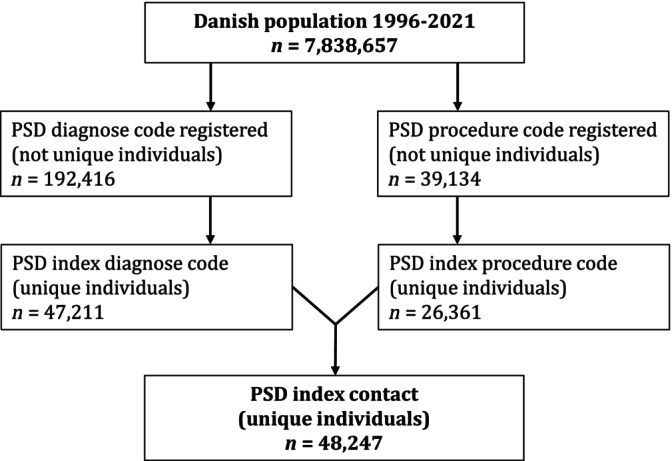
Flowchart of pilonidal sinus disease (PSD) diagnosis and procedure code numbers in the Danish population from 1996 to 2021.

We identified 48 247 individuals with either a diagnosis or a procedure code specific to PSD from 1996 to 2021 in a general population of 7 838 657 unique individuals (50% female/50% male) in the same period (Figure 1). Overall, 72% of patients with PSD were male and 28% were female. The proportion of male patients varied from 69% to 73% over the period (Table 1).

**TABLE 1:** Baseline characteristics of pilonidal sinus disease patients overall and in each period from 1996 to 2021.

Characteristic	Overall (*N* = 48 247)	Years				
		1996–2000 (*n* = 7321)	2001–2005 (*n* = 8483)	2006–2010 (*n* = 8784)	2011–2015 (*n* = 9940)	2016–2021 (*n* = 13 719)
Total						
Age (years), median (IQR)	25 (20, 34)	26 (21, 33)	26 (20, 34)	25 (20, 35)	24 (19, 35)	24 (20, 34)
Sex						
Female	28%	31%	27%	27%	28%	29%
Male	72%	69%	73%	73%	72%	71%
Diagnostic type						
Abscess‐forming	53%	58%	54%	51%	53%	52%
Nonabscess forming	47%	42%	46%	49%	47%	48%
Missing (*n*)	19	5	<5	<5	14	<5
Male patients						
Age (years), median (IQR)	26 (20, 35)	27 (22, 34)	26 (21, 35)	26 (20, 35)	25 (20, 36)	25 (20, 34)
Diagnostic type						
Abscess‐forming	49%	55%	50%	48%	48%	46%
Nonabscess forming	51%	45%	50%	52%	52%	54%
Missing (*n*)	15	<5	<5	<5	12	<5
Female patients						
Age (years), median (IQR)	23 (18, 32)	23 (19, 30)	24 (19, 32)	24 (19, 34)	22 (18, 32)	23 (18, 31)
Diagnostic type						
Abscess‐forming	64%	64%	64%	61%	65%	65%
Nonabscess forming	36%	36%	36%	39%	35%	35%
Missing (*n*)	<5	<5	<5	<5	<5	<5

Abbreviation: IQR, interquartile range.

Female patients were younger at the time of index contact than male patients. In male patients, the median age at index contact decreased from 27 years [interquartile range (IQR) 22–34 years] in 1996–2000 to 25 years (IQR 20–34 years) in 2016–2021. The median age at index contact remained constant over time in female patients, at around 23 years (IQR 18–32 years) (Table 1).

The distribution between disease types differed between male and female patients. Among male patients, 55% presented with abscess‐forming PSD at index contact in 1996–2000 compared with only 46% in 2015–2021. Female patients presented with abscess‐forming PSD in 64% of cases, which did not change over the study period (Table 1).

### Incidence rates

**FIGURE 2:**
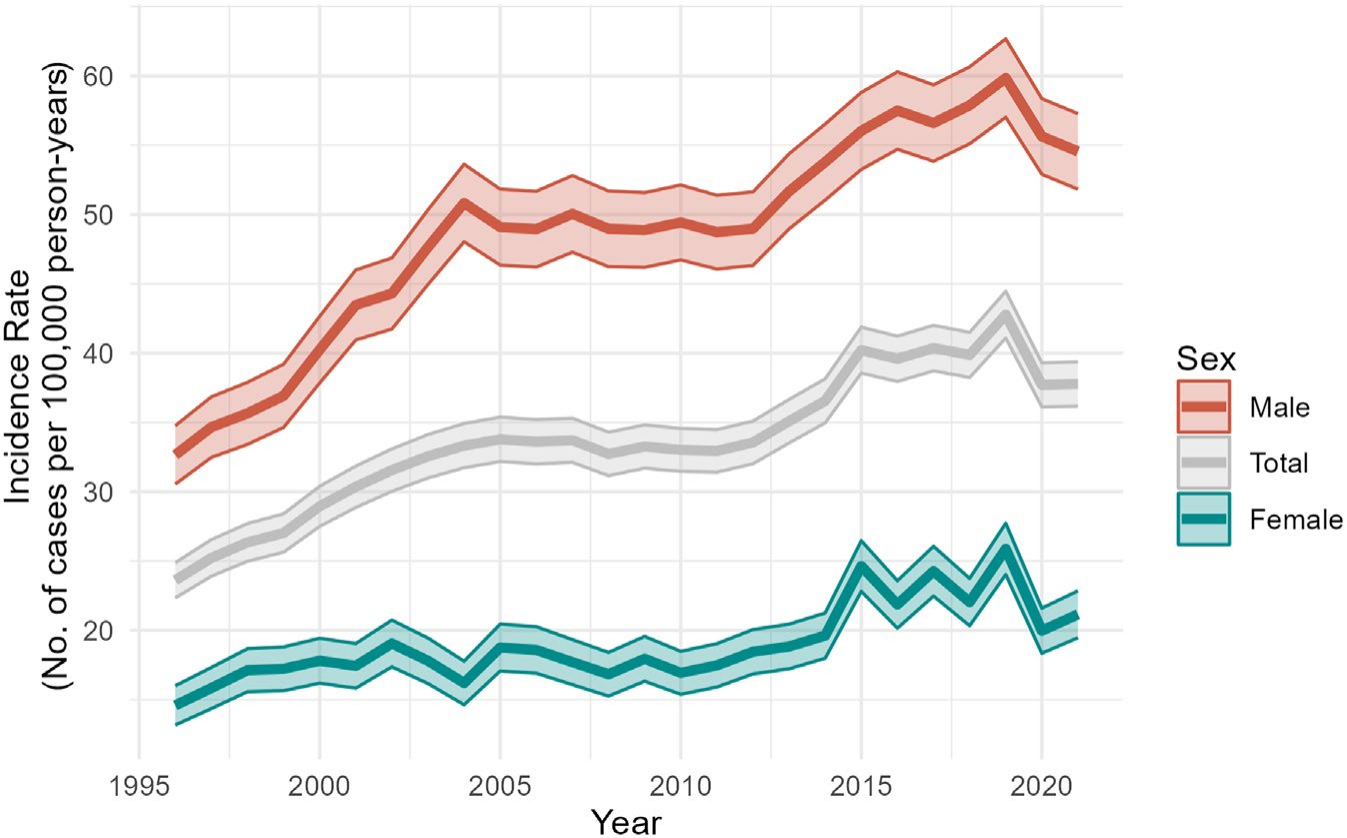
Annual incidence of pilonidal sinus disease per 100 000 person‐years from 1996 to 2021 in male and female patients.

Overall, the incidence has increased in both genders from 1996 to 2021 (Figure 2). Comparing the 1996–2000 period with the 2016–2021 period, the overall age‐standardized incidence rate of PSD increased from 26.1 to 39.6/100 000 person‐years (PY), corresponding to an IRR of 1.52 (95% CI 0.78–2.94) and an IRD of 13.5 (95% CI 12.83–14.15) (Table 2). The observed increase was more pronounced among male patients, for whom the incidence rate of PSD increased from 35.8 to 56.9/100 000 PY, corresponding to an IRR of 1.59 (95% CI 0.52–4.89) and an IRD of 21.0 (95% CI 19.9–22.15). Among female PSD patients, the incidence rate increased from 16.4 to 22.5/100 000 PY, corresponding to an IRR of 1.4 (95% CI 0.68–2.76) and an IRD 6.0 (95% CI 5.34–6.75) (Table 2).

**TABLE 2:** Age‐adjusted incidence rates, incidence rate ratios and incidence rate difference per 100 000 person‐years.

	Incidence rate/100 000 PY		Incidence rate ratio		Incidence rate difference	
	IR	95% CI	IRR	95% CI	IRD	95% CI
Total						
Year						
1996–2000	26.07	25.47–26.67	1.00	Reference	0.00	Reference
2001–2005	32.2	31.51–32.90	1.24	0.62–2.47	6.14	5.44–6.83
2006–2010	33.14	32.44–33.84	1.27	0.63–2.56	7.07	6.37–7.77
2011–2015	35.59	34.89–36.30	1.37	0.68–2.76	9.52	8.82–10.23
2016–2021	39.56	38.89–40.22	1.52	0.78–2.94	13.49	12.83–14.15
Male patients						
Year						
1996–2000	35.79	34.80–36.79	1.00	Reference	0.00	Reference
2001–2005	46.94	45.75–48.12	1.31	0.40–4.29	11.15	9.96–12.33
2006–2010	49.09	47.88–50.30	1.37	0.41–4.61	13.3	12.09–14.51
2011–2015	51.71	50.51–52.92	1.44	0.43–4.82	15.92	14.72–17.13
2016–2021	56.82	55.69–57.94	1.59	0.52–4.89	21.03	19.9–22.15
Female patients						
Year						
1996–2000	16.43	15.75–17.11	1.00	Reference	0.00	Reference
2001–2005	17.77	17.04–18.49	1.08	0.52–2.24	1.34	0.61–2.07
2006–2010	17.52	16.81–18.24	1.07	0.52–2.18	1.1	0.38–1.87
2011–2015	19.78	19.04–20.51	1.2	0.58–2.51	3.35	2.61–4.08
2016–2021	22.47	21.77–23.17	1.37	0.68–2.76	6.04	5.34–6.75

Abbreviations: CI, confidence interval. PY, person‐years. IR, incidence rate. IRR, incidence rate ratio. IRD, incidence rate difference.

### Age‐specific incidence

**FIGURE 3:**
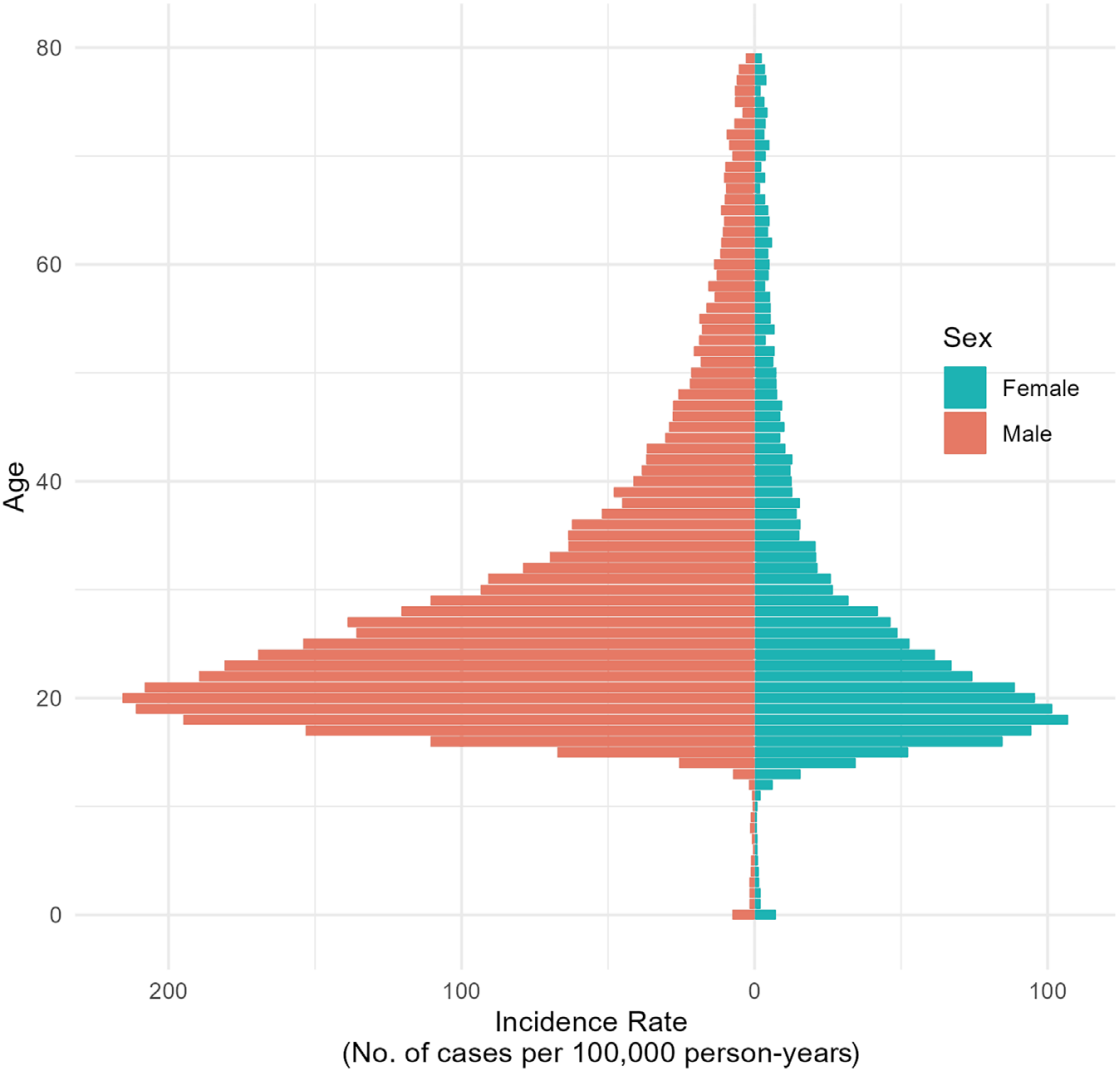
Age‐specific crude incidence rate of pilonidal sinus disease by sex. This population pyramid displays the age‐specific crude incidence rates of pilonidal sinus disease within the cohort, stratified by age and sex. The highest age‐specific incidence is found among 20‐year‐old men and 18‐year‐old women, respectively.

**FIGURE 4:**
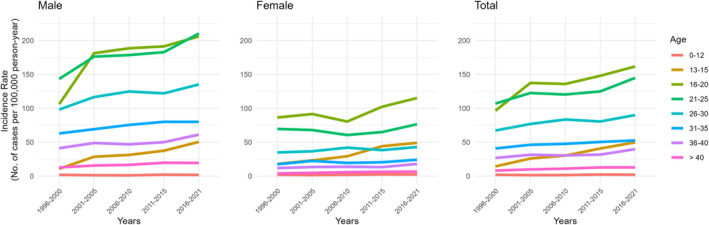
Age‐specific incidence of pilonidal sinus disease per 100 000 person‐years from 1996 to 2021 in male and female patients.

The highest incidence was observed among 20‐year‐old men, with a rate of 216.9/100 000 PY (95% CI 207.2–226.6). Among female patients, the highest incidence was observed among 18‐year‐olds, with a rate of 107.9/100 000 PY (95% CI 100.8–115.0) (Figure 3). Generally, we observed that the incidence in the early teenage years was higher among female than male patients, but shifted towards a higher incidence in male patients at the age of 15 years and above (Figure 3). Moreover, the most substantial difference in incidence over time occurred among men and boys aged 16–20 years at index, with an IRD of 99.9/100 000 PY (95% CI 88.74–111.14). In contrast, the highest difference in incidence among female patients occurred at an earlier age, with an IRD of 31.1/100 000 PY (95% CI 24.17–38.12) for those aged 13–15 years. Additionally, when examining the IRR across different age groups, we found that 13–15‐year‐olds had the highest IRR in both sexes [female IRR 2.73 (95% CI 2.12–3.53), male IRR 4.66 (95% CI 3.42–6.35)] (Figure 4, Table S2).

## DISCUSSION

To our knowledge, this is the first nationwide population‐based study on time trends in the incidence of PSD. It cements the increase in incidence from 1996 to 2021 in both sexes and identifies the main drivers as male patients and adolescents. Furthermore, it shows that the most substantial increase occurred among adolescents.

### Time trends in incidence

Our findings are thus on a par with the previous findings from the German military registries and inpatient admissions for treatment of PSD [7, 8, 9]. Our study extends existing knowledge by examining the trend using a population‐based approach that includes both inpatient and outpatient contacts. This method is likely to provide a more accurate estimation of incidence, age at debut and sex distribution than previous studies from more selected settings. Moreover, our data provide an overview of the incidence across all age groups, revealing a disease that is dominant in late adolescence but with the most significant increase in incidence occurring in early adolescence.

The increasing incidence is a topic for speculation. In Denmark, access to healthcare has not changed during the study period, and no policy has been changed regarding the treatment of PSD [11]. The awareness of PSD increased around 2008 due to an awareness in the Danish Surgical Society of the surgical failure rates in PSD patients, often handled by untrained hands. This led to the specification of procedure codes and national workshops on Bascom's pit‐pick and cleft lift surgery, which may be part of the explanation. However, PSD was a commonly known condition in general surgery both before and after 2010, and a change in procedure code cannot account for the rise alone, especially when considering that the same surge has been seen in other countries. Lifestyle factors, including a more sedentary lifestyle, smoking or an increase in body mass index, have been proposed as part of the explanation as they are considered risk factors for developing PSD [17, 18]. However, studies exploring these factors have failed to establish a connection [18, 19, 20, 21]. As short, loose hairs have been found to dominate the content of pilonidal cysts [2, 22, 23], the increase in incidence may be due to a change in hairstyle towards shorter, machine‐trimmed hair. As men and boys are more prone to this hairstyle and have been shown to have stronger axial hair force [24], this may explain the sex distribution. Whether or not changes in hairstyle or cutting techniques correlate with our findings remains unknown. As stronger axial hair is correlated with patients' ethnicity [25], part of the explanation for the rise in incidence may be found in the changing composition of the population in Denmark, as immigration from both non‐Western and Western countries increased in Denmark during the study period [26]. The significance of ethnicity in the development of PSD has not yet been fully clarified. However, several studies suggest that it is a significant factor [10, 27], and a higher incidence has been found among Turkish and Greek soldiers [22, 28] than in northern European studies with populations comparable to the one in our study [7, 8, 17]. However, military studies represent highly selected patient groups consisting mainly of young men.

### Age at index contact for PSD

Compared with previous studies, we found a higher median age at first contact [17, 28, 29, 30]. This may be due to our large cohort, which includes all age groups and is thus not restricted to specific age groups or a defined study population. Moreover, our findings of the highest age‐specific incidence at 18 and 20 years of age for women and men, respectively, confirm that PSD is a disease dominant in late adolescence, despite the overall age distribution raising the median age (Figure 3).

### Adolescent PSD

Notably, PSD is increasing considerably among 13–15‐year‐olds, though the absolute numbers indicate that male patients aged 16–20 are most frequently encountered in clinical settings. Additionally, the relative rise in incidence has been substantial in the 13–15‐year age group for both sexes. Even if the median age for female PSD is stable, the incidence is rising considerably in young age groups. This may be due to the earlier debut of puberty in Denmark, as puberty is known to be a factor in development of PSD [21, 29, 31, 32]. Although we have no proof that the early debut of PSD causes a more severe disease, young age has been shown to increase the rate of recurrence [30]. As recurrent disease is considered, by most, as a more advanced disease [4, 33], the declining age at debut is clinically relevant in the treatment of PSD. Young age is a factor that should be taken into account in the treatment of PSD to limit the risk of recurrence, and the consequences of early debut of PSD for recurrence and disease manifestation is an important aspect for future research.

### Female PSD

We found female PSD patients to have an earlier disease debut than male patients. The driver of this difference seems to be a distinct age‐specific incidence pattern, with a higher incidence among female patients in the early teenage years. Furthermore, the proportion of female patients in our study is higher than the 21% reported in a meta‐analysis on global sex differences in PSD [10]. However, as observed in our study, female patients are more likely to present with abscess‐forming PSD at the time of diagnosis than are male patients. Since most studies on surgical treatment of PSD are primarily focused on nonabscess‐forming cases, these studies may have a falsely high proportion of male patients.

### Disease type

Most PSD studies focus primarily on elective treatment of both abscess and nonabscess‐forming PSD in nonacute stages, disregarding this key difference in manifestation. The actual burden of abscess‐forming PSD compared with nonabscess‐forming PSD was previously unknown. In studies conducted in German military hospitals, acute/abscess‐forming PSD accounted for 24%–36% of PSD treated there. However, military cohorts are highly selected in terms of sex and age [18, 29, 30]. In a 1994 Norwegian study, 47% of participants were found to have a history of previous acute abscesses, but this patient population consisted of patients referred to treatment of chronic disease and thus did not include the assumed proportion of patients with pilonidal abscess who were cured by incision and drainage alone (or previously by excision in the acute stage) [17]. Furthermore, in some countries, incision treatments are administered by general practitioners outside the hospital system and are thus not included in studies/registries [34]. These studies may, therefore, have a falsely low proportion of abscess‐forming PSD compared with our study, where incision treatment is solely performed within the hospital system and with our cohort consisting of all ages and both sexes.

### Strengths and limitations

Denmark's hospital and registration system enhances the robustness of our study, as all hospitals are subject to rules for reporting diagnostic and procedure codes to the DNPR for both inpatient admissions and outpatient visits. Even if the completeness of PSD registration is unknown, conditions that necessitate hospital encounters are consistently recorded, and the overall completeness of diagnoses in the DNPR has been studied elsewhere and found to be higher than in the clinical registries [12].

Furthermore, there is a likelihood of underreporting of PSD in our study, as some patients may choose never to seek treatment for PSD. However, patients are probably more inclined to seek medical care in a tax‐based healthcare system such as the Danish system. Moreover, our study is the first to validate the diagnostic coding of PSD, finding a sufficiently high positive predictive value to support the use of Danish registries in PSD research.

Our study also has certain limitations. Before 2003, private hospitals could have neglected to report procedures. However—as already mentioned—private hospitals and clinics are few, and we observe no substantial rise in incidence in 2003. The changes to procedure codes in 2010 did not affect the reported incidence either. The change in the reporting system in the DNPR in 2019 entailed an administrative discharge of all patients and a simultaneous start of new admissions, which had a great impact on data registration in the latter part of our study period. As we observe a sudden rise in incidence followed by a drop during the same period, we do not expect the decrease to be a sign of a turning point in the rise in incidence but rather a result of the administrative change. Finally, it remains uncertain whether our findings represent a global trend, as the incidence of PSD appears to be subject to geographical and ethnic differences [7, 10, 28, 35].

## CONCLUSION

This register‐based cohort study provides an overview of the change in the incidence of PSD during the last decades in both sexes and across all age groups in a Danish nationwide setting. Incidence has increased in both sexes, notably among young men. A recent multicentre study showed that most PSD patients experience multiple recurrences and a significant impact on daily activities [4, 36]. These findings need to be explored in population‐based data. Further, we know nothing about the possible long‐term effects of PSD and multiple surgeries in this region and how they impact these patients. It may be time for the surgical community to acknowledge the growing number of young people affected by PSD and increase efforts to improve outcomes through research, training and organization of surgical volume.

## AUTHOR CONTRIBUTIONS

**Ida Kaad Faurschou:** Conceptualization; investigation; funding acquisition; writing – original draft; methodology; validation; visualization; formal analysis; software; project administration; writing – review and editing. **Rune Erichsen:** Conceptualization; supervision; writing – review and editing; methodology; formal analysis; investigation; data curation; funding acquisition; validation. **Dietrich Doll:** Writing – review and editing; supervision; resources; investigation. **Susanne Haas:** Conceptualization; methodology; validation; writing – review and editing; supervision; funding acquisition; investigation; project administration.

## FUNDING INFORMATION

I. K. Faurschou is supported by a scholarship from Aarhus University.

## CONFLICT OF INTEREST STATEMENT

The author(s) have no affiliations or financial involvement with any organization or entity with financial interest.

## ETHICS STATEMENT

In accordance with Danish legislation, registry‐based studies do not necessitate informed consent or approval from an ethics committee. The study was reported to Aarhus University and received the registration number 2624.

## Supporting information


Table S1.

Table S2.

## Data Availability

According to Danish legislation, data used for this study cannot be shared or made available to other parties. Researchers from certified Danish research institutions who wish to access the databases used in this study may email Statistics Denmark (dst@dst.dk).
