# Supplementary material for: Time trends in incidence of pilonidal sinus disease from 1996 to 2021: A Danish population‐based cohort study
Source: Colorectal Dis. 2024 Nov 3;27(1):e17227. doi: 10.1111/codi.17227 (PMC11683168; doi:10.1111/codi.17227)
Supplement: Supplementary file 1 — Table S1. [file CODI-27-0-s002.docx]

**Supplementary Table 1:** Overview of the diagnosis and procedure codes used in the Danish National Patient Registry (DNPR) from 1971 to today to register pilonidal sinus disease (PSD).

| **Diagnosis code** | **ICD-8** | **ICD-10** | **Disease type** |
| --- | --- | --- | --- |
|  | (1971-1993) | (1994-today) |  |
| PSD | 685.09 | L05 | Undefined |
| PSD without abscess | 685 | L059 | Non-abscess forming |
| PSD with abscess | 685.01 | L050 | Abscess-forming |
| **Surgical procedure code** | **Danish surgical codes** | **NOMESCO Classification of Surgical Procedures** (1996- 2010^1^, 2010 - today^2^) |  |
|  | (1971-1995) |  |  |
| Incision of pilonidal cyst | 46920 | KQBA15^1^, KQBA10B^2^ | Abscess-forming |
| Excision of pilonidal cyst | 46940 |  | Non-abscess forming |
| Excision of pilonidal cyst on truncus |  | KQBE15^1^, KQBE10C^2^ | Non-abscess forming |
| Pit-pick operation am Bascom for pilonidal cyst |  | KQBE10CC^2^ | Non-abscess forming |
| Cleft-lift operation am Bascom for pilonidal cyst |  | KQBE10CD^2^ | Non-abscess forming |
| Surgery for pilonidal cyst am Lord-Millar |  | KQBE10CE^2^ | Non-abscess forming |
| Excision of pilonidal cyst with other plastic |  | KQBE10CF^2^ | Non-abscess forming |
| Wide excision of pilonidal cyst with primary closure in the midline |  | KQBE10CA^2^ | Non-abscess forming |
| Wide excision of pilonidal cyst with Sec healing (open surgery) |  | KQBE10CB^2^ | Non-abscess forming |

Abbreviation: am, ad modum. ICD8/10, World Health Organisation’s International Classification of Diseases 8^th^ /10^th^ revision. NOMESCO, The Nordic Medico-Statistical Committee.
