# Supplementary material for: Time trends in incidence of pilonidal sinus disease from 1996 to 2021: A Danish population‐based cohort study
Source: Colorectal Dis. 2024 Nov 3;27(1):e17227. doi: 10.1111/codi.17227 (PMC11683168; doi:10.1111/codi.17227)
Supplement: Supplementary file 2 — Table S2. [file CODI-27-0-s001.docx]

**Supplementary Table 2:** Time trend in incidence stratified by age group in each period from 1996-2021.

|  |  | **Male** | | | | | |  | **Female** | | | | | |
| --- | --- | --- | --- | --- | --- | --- | --- | --- | --- | --- | --- | --- | --- | --- |
| **Age group** | **Years** | **IR/100,000PY** | **95%CI** | **IRR** | **95%CI** | **IRD** | **95%CI** |  | **IR/100,000PY** | **95%CI** | **IRR** | **95%CI** | **IRD** | **95%CI** |
| 0-12 | 1996-2000 | 1.9 | 1.32-2.48 | 1 | Reference | 0 | Reference |  | 2 | 1.39-2.62 | 1 | Reference | 0 | Reference |
|  | 2001-2005 | 1.37 | 0.89-1.85 | 0.72 | 0.45-1.15 | -0.53 | -1.29-0.22 |  | 1.58 | 1.05-2.11 | 0.79 | 0.5-1.24 | -0.42 | -1.23-0.39 |
|  | 2006-2010 | 1.17 | 0.72-1.62 | 0.61 | 0.38-1 | -0.73 | -1.47-0 |  | 1.98 | 1.38-2.58 | 0.99 | 0.64-1.52 | -0.02 | -0.88-0.84 |
|  | 2011-2015 | 2.13 | 1.52-2.75 | 1.12 | 0.74-1.71 | 0.23 | -0.62-1.08 |  | 2.58 | 1.89-3.28 | 1.29 | 0.86-1.94 | 0.58 | -0.35-1.51 |
|  | 2016-2021 | 1.77 | 1.25-2.29 | 0.93 | 0.61-1.42 | -0.13 | -0.92-0.65 |  | 2.42 | 1.79-3.04 | 1.21 | 0.81-1.8 | 0.41 | -0.46-1.29 |
| 13-15 | 1996-2000 | 10.84 | 7.71-13.97 | 1 | Reference | 0 | Reference |  | 17.96 | 13.84-22.07 | 1 | Reference | 0 | Reference |
|  | 2001-2005 | 28.61 | 23.82-33.4 | 2.64 | 1.89-3.69 | 17.77 | 12.05-23.49 |  | 23.16 | 18.73-27.6 | 1.29 | 0.96-1.74 | 5.21 | -0.84-11.26 |
|  | 2006-2010 | 31.29 | 26.57-36 | 2.89 | 2.08-4 | 20.45 | 14.79-26.11 |  | 29.26 | 24.58-33.94 | 1.63 | 1.23-2.16 | 11.3 | 5.07-17.54 |
|  | 2011-2015 | 37.51 | 32.28-42.73 | 3.46 | 2.51-4.77 | 26.67 | 20.58-32.76 |  | 44.14 | 38.34-49.95 | 2.46 | 1.89-3.2 | 26.19 | 19.07-33.31 |
|  | 2016-2021 | 50.48 | 44.91-56.06 | **4.66** | **3.42-6.35** | 39.65 | 33.25-46.04 |  | 49.1 | 43.47-54.73 | **2.73** | **2.12-3.53** | **31.14** | **24.17-38.12** |
| 16-20 | 1996-2000 | 106.12 | 98.92-113.32 | 1 | Reference | 0 | Reference |  | 86.42 | 79.78-93.06 | 1 | Reference | 0 | Reference |
|  | 2001-2005 | 181.27 | 171.5-191.03 | 1.71 | 1.57-1.86 | 75.14 | 63.01-87.27 |  | 91.61 | 84.51-98.71 | 1.06 | 0.95-1.18 | 5.19 | -4.52-14.91 |
|  | 2006-2010 | 188.53 | 179.22-197.83 | 1.78 | 1.63-1.93 | 82.4 | 70.64-94.17 |  | 80.51 | 74.27-86.76 | 0.93 | 0.84-1.04 | -5.9 | -15.02-3.21 |
|  | 2011-2015 | 191.35 | 182.43-200.28 | 1.8 | 1.66-1.96 | 85.23 | 73.77-96.7 |  | 102.31 | 95.62-109.01 | 1.18 | 1.07-1.31 | 15.9 | 6.47-25.33 |
|  | 2016-2021 | 206.06 | 197.48-214.64 | 1.94 | 1.79-2.1 | **99.94** | **88.74-111.14** |  | 115.15 | 108.58-121.73 | 1.33 | 1.21-1.47 | 28.73 | 19.39-38.08 |
| 21-25 | 1996-2000 | 143.31 | 135.61-151 | 1 | Reference | 0 | Reference |  | 69.63 | 64.19-75.07 | 1 | Reference | 0 | Reference |
|  | 2001-2005 | 176.25 | 167.07-185.42 | 1.23 | 1.14-1.33 | 32.94 | 20.96-44.91 |  | 67.97 | 62.22-73.72 | 0.98 | 0.87-1.1 | -1.66 | -9.57-6.26 |
|  | 2006-2010 | 178.55 | 169.13-187.97 | 1.25 | 1.16-1.34 | 35.24 | 23.08-47.41 |  | 60.64 | 55.08-66.21 | 0.87 | 0.77-0.98 | -8.99 | -16.77--1.21 |
|  | 2011-2015 | 182.81 | 173.97-191.66 | 1.28 | 1.19-1.37 | 39.51 | 27.79-51.23 |  | 64.98 | 59.61-70.34 | 0.93 | 0.83-1.05 | -4.65 | -12.29-2.98 |
|  | 2016-2021 | 210.42 | 202.16-218.67 | 1.47 | 1.37-1.57 | 67.11 | 55.83-78.4 |  | 76.53 | 71.44-81.61 | 1.1 | 0.99-1.22 | 6.89 | -0.55-14.34 |
| 26-30 | 1996-2000 | 98.22 | 92.08-104.35 | 1 | Reference | 0 | Reference |  | 34.88 | 31.15-38.62 | 1 | Reference | 0 | Reference |
|  | 2001-2005 | 116.62 | 109.71-123.53 | 1.19 | 1.09-1.29 | 18.41 | 9.17-27.64 |  | 36.65 | 32.75-40.55 | 1.05 | 0.9-1.22 | 1.77 | -3.63-7.17 |
|  | 2006-2010 | 124.84 | 117.22-132.46 | 1.27 | 1.16-1.39 | 26.62 | 16.84-36.4 |  | 41.99 | 37.56-46.42 | 1.2 | 1.04-1.4 | 7.11 | 1.31-12.9 |
|  | 2011-2015 | 122.07 | 114.46-129.68 | 1.24 | 1.14-1.36 | 23.86 | 14.08-33.63 |  | 38.35 | 34.04-42.65 | 1.1 | 0.94-1.28 | 3.47 | -2.23-9.16 |
|  | 2016-2021 | 135.22 | 128.56-141.87 | 1.38 | 1.27-1.49 | 37 | 27.95-46.05 |  | 42.94 | 39.11-46.77 | 1.23 | 1.07-1.42 | 8.06 | 2.71-13.41 |
| 31-35 | 1996-2000 | 63 | 58.25-67.75 | 1 | Reference | 0 | Reference |  | 17.47 | 14.91-20.04 | 1 | Reference | 0 | Reference |
|  | 2001-2005 | 69.19 | 64.05-74.34 | 1.1 | 0.99-1.22 | 6.2 | -0.81-13.2 |  | 22.46 | 19.47-25.44 | 1.29 | 1.05-1.57 | 4.98 | 1.05-8.92 |
|  | 2006-2010 | 75.51 | 69.97-81.05 | 1.2 | 1.08-1.33 | 12.51 | 5.21-19.81 |  | 19.44 | 16.62-22.26 | 1.11 | 0.9-1.37 | 1.97 | -1.85-5.78 |
|  | 2011-2015 | 80.09 | 74.06-86.12 | 1.27 | 1.14-1.41 | 17.09 | 9.42-24.77 |  | 20.48 | 17.42-23.54 | 1.17 | 0.95-1.45 | 3.01 | -0.99-7 |
|  | 2016-2021 | 80.07 | 74.65-85.49 | 1.27 | 1.15-1.41 | 17.07 | 9.86-24.28 |  | 24.14 | 21.11-27.17 | 1.38 | 1.14-1.68 | 6.67 | 2.7-10.64 |
| 36-40 | 1996-2000 | 41.21 | 37.17-45.25 | 1 | Reference | 0 | Reference |  | 11.89 | 9.68-14.11 | 1 | Reference | 0 | Reference |
|  | 2001-2005 | 48.88 | 44.69-53.08 | 1.19 | 1.04-1.35 | 7.68 | 1.86-13.5 |  | 13.44 | 11.2-15.68 | 1.13 | 0.88-1.45 | 1.55 | -1.6-4.7 |
|  | 2006-2010 | 46.82 | 42.59-51.05 | 1.14 | 0.99-1.3 | 5.61 | -0.24-11.46 |  | 13.82 | 11.49-16.15 | 1.16 | 0.9-1.49 | 1.93 | -1.29-5.14 |
|  | 2011-2015 | 50.08 | 45.58-54.57 | 1.22 | 1.06-1.39 | 8.87 | 2.83-14.91 |  | 13.28 | 10.96-15.6 | 1.12 | 0.87-1.44 | 1.39 | -1.82-4.59 |
|  | 2016-2021 | 61.19 | 56.41-65.97 | 1.48 | 1.31-1.68 | 19.98 | 13.72-26.24 |  | 17.97 | 15.36-20.58 | 1.51 | 1.19-1.91 | 6.08 | 2.66-9.5 |
| > 40 | 1996-2000 | 12.9 | 11.97-13.83 | 1 | Reference | 0 | Reference |  | 3.93 | 3.45-4.42 | 1 | Reference | 0 | Reference |
|  | 2001-2005 | 15.6 | 14.6-16.6 | 1.21 | 1.1-1.33 | 2.7 | 1.34-4.07 |  | 4.78 | 4.25-5.31 | 1.21 | 1.03-1.43 | 0.84 | 0.12-1.56 |
|  | 2006-2010 | 16.63 | 15.64-17.63 | 1.29 | 1.17-1.42 | 3.74 | 2.37-5.1 |  | 5.8 | 5.23-6.37 | 1.47 | 1.26-1.73 | 1.87 | 1.12-2.62 |
|  | 2011-2015 | 19.76 | 18.7-20.82 | 1.53 | 1.4-1.68 | 6.86 | 5.45-8.27 |  | 6.39 | 5.81-6.98 | 1.63 | 1.39-1.9 | 2.46 | 1.7-3.22 |
|  | 2016-2021 | 19.45 | 18.52-20.39 | 1.51 | 1.38-1.64 | 6.56 | 5.24-7.87 |  | 6.64 | 6.12-7.17 | 1.69 | 1.46-1.96 | 2.71 | 1.99-3.43 |
|  |  |  |  |  |  |  |  |  |  |  |  |  |  |  |

Abbreviation: IR, Incidence Rate. IRR, Incidence Rate Ratio. IRD, Incidence Rate Difference. PY, Person-years.
